# Crural Closure improves Outcomes of Magnetic Sphincter Augmentation in GERD patients with Hiatal Hernia

**DOI:** 10.1038/s41598-018-24322-1

**Published:** 2018-05-09

**Authors:** Katrin Schwameis, Milena Nikolic, Deivis G. Morales Castellano, Ariane Steindl, Sarah Macheck, M. Riegler, Ivan Kristo, Barbara Zörner, Sebastian F. Schoppmann

**Affiliations:** Department of Surgery, Division of General Surgery, Medical University of Vienna, Waehringer Guertel 18-20, 1090 Vienna, Austria

## Abstract

Magnetic sphincter-augmentation (MSA) has been proven effective in the treatment of GERD. No consensus exists on whether crural closure should be performed. Our aim was to assess the impact of cruroplasty on reflux-control and quality of life. MSA-Patients treated between 03/2012-03/2017 were classified into those without hiatal hernia (“NHH”), those post-MSA (NHR) and those post-MSA/hiatal repair (HR). GERD-symptoms, PPI-intake, GERD-Health-related-Quality-of-Life (GERD-HRQL) and Alimentary Satisfaction were assessed. Sixty-eight patients underwent MSA, 26 patients had additional crural closure. PH-monitoring was negative in 80% of HR, 73% of NHR and 89% of NHH-patients. GERD-HRQL-total scores decreased significantly in all groups (*p* < 0.001). Alimentary satisfaction was 8/10 in HR/NHH and 10/10 in NHR-patients. Satisfaction with heartburn relief was high (HR: 96%, NR: 95%, NHH: 94%) as was the elimination of PPI-intake (HR/NHH: 87%, NR: 86%). Heartburn and regurgitations were eliminated in 100% of HR, 88% and 94% of NHR and 87% and 91% of NHH-patients. Endoscopic dilatation or device explantation was performed in 3% each. MSA leads to significant symptom relief, increased quality of life and alimentary satisfaction with low perioperative morbidity. Cruroplasty tends to result in better reflux control and symptom relief than exclusive MSA without increasing dysphagia rates.

## Introduction

Gastroesophageal Reflux Disease (GERD) is one of the most common gastrointestinal disorders affecting up to 25% of the population in the Western World^1^. The surgical gold standard in the treatment of GERD is the laparoscopic fundoplication^1–3^. Typical side effects include gas bloat syndrome, a reduced ability to vomit and belch and dysphagia. A further deterrent of this procedure is the extensive alteration of the anatomy making it difficult to re-operate if needed^4^. A recently introduced alternative with possibly less side effects is the magnetic sphincter augmentation (MSA) with a small device consistent of magnetic beats (LINX® Reflux Management System; Torax Medical, Maple Grove, MN). The device is placed laparoscopically around the gastroesophageal junction to augment the barrier function of the lower esophageal sphincter (LES), suppressing reflux episodes while enabling the physiological functions of the LES uninhibited. This can be accomplished with either focused or full dissection^5^. The former minimally invasive procedure keeps the possibility of further anti-reflux surgery open, if needed, by maintaining the esophageal and hiatal anatomy^4,6^.

Former studies showed that the implantation of LINX was safe and efficient in reducing the dependence on proton pump inhibitors (PPIs), improving GERD-specific quality of life while leading to a low rate of side effects^3,6,7^. While MSA was initially limited to patients with small or no hiatal hernias Rona K. *et al*. recently reported their encouraging outcomes of MSA in patients with hiatal hernias up to 7 cm. They showed that these patients had similar postoperative symptom relief, decreased PPI requirement, GERD-HRQL scores and dysphagia rates as patients with smaller hernias^8^.

As described previously hiatal dissection followed by suture crural repair is a crucial step in the process of laparoscopic anti-reflux surgeries to prevent hiatal herniation^9^.

However, no consensus exists on whether additional hiatal repair should be routinely performed in patients undergoing sphincter augmentation. Currently, to our knowledge, no study has been published comparing outcomes of patients after exclusive MSA with those who have had MSA and additional crural closure.

The aim of this study was to assess if hiatal repair in MSA patients significantly influences postoperative outcomes including reflux control and dysphagia rate.

## Methods

A retrospective chart review was performed to identify all GERD patients that underwent anti-reflux surgery (ARS) between 03/2012 and 03/2017 at our institution. Of these 331 cases (100%), patients treated with laparoscopic Nissen fundoplication (n = 263; 80%) were excluded from the study. This led to a final study population of 68 patients (20% of all ARS) that had undergone laparoscopic magnetic sphincter augmentation (MSA).

The initial 37 patients had received exclusive magnetic sphincter augmentation and were followed by a consecutive series of 31 patients that had undergone MSA and crural closure. Based on the presence of a hiatal hernia (HH) and performed treatment patients were classified into 3 groups: “No hiatal hernia” (NHH), “Hiatal Repair” (HR) and “No Hiatal Repair” (NHR) group.

A detailed review of a prospectively created database was performed to obtain preoperative clinical and radiological data, histological results as well as outcome parameters.

Furthermore, a standardized interview was performed by the same physician assessing postoperative gastrointestinal symptoms, proton pump inhibitor intake (PPI), GERD-Health-related-Quality-of-Life (GERD-HRQL) and Alimentary Satisfaction (AS). Overall alimentary tract comfort was rated from 0–10. A score of 10 indicated complete satisfaction, and a score of 0 indicated an intolerable alimentary function^10^. Patients served as their own control when comparing pre- and postoperative scores of validated questionnaires including the GERD Health-Related Quality-of-Life (HRQL)^11–13^. The frequency and severity of postoperative dysphagia was assessed using the classification of Saeed *et al*.^14^. Patients with longer follow-up times were scheduled for postoperative esophageal functioning testing (EFT) including the performance of a high-resolution manometry and impedance-pH-metry.

This study was approved by the Institutional Review Board of the Medical University of Vienna, Austria. All methods were carried out in accordance with relevant guidelines and regulations. An individual informed consent was not acquired, due to study design and national regulations.

### Preoperative evaluation

Preoperative diagnostics included a standardized interview, the performance of an upper GI endoscopy, a video esophagram and esophageal functioning testing consistent of a high-resolution manometry (HRM) and a 24-hour-Impedance-pH-metry.

All patients underwent preoperative assessment by high-resolution manometry (Sandhill BioView; Sandhill Zvu; Medtronic ManoScan). Manometric findings were reported in accordance to the Chicago classification v3.0.

Patients were off proton pump inhibitors for 14 days prior to pH-monitoring. All patients underwent an ambulatory continuous 24-hour esophageal impedance-pH-monitoring with a transnasal catheter (Sandhill ComforTec Z/pH ZAN-BS-01/ZAN-BG-44). The pH-probes were positioned on the basis of manometry findings 5 cm above the upper border of the lower esophageal sphincter (LES) as previously described^15^. Patients were instructed to precisely document their food and fluid intakes in a diet diary. Analysis of impedance-pH-results included the total number of reflux episodes, the percentage time pH < 4 in total, in upright and supine position and postprandial. An abnormal pH-test was based on the number of reflux episodes (normal < 73 episodes/24 hours) and the total percentage time pH < 4 (normal < 4.2%). Additionally, a symptom correlation analysis was performed.

### Surgery

All procedures were performed by the same surgeon using standard surgical techniques as described previously^16,17^. The surgical approach was laparoscopic in all cases and there were no conversions to open surgery. Briefly, after mobilization of the esophagogastric junction the adequate ring size was measured with the sizing tool and the magnetic device was wrapped around the lower end of the lower esophageal sphincter. All procedures were standardized regarding the surgeon´s and patient’s positions (anti-Trendelenburg), the trocar sites and the used instruments.

After the performance of our initial 37 magnetic sphincter augmentations a consecutive series of 31 patients followed that received additional hiatal repair. These procedures were accomplished by hiatal dissection and crural closure using non-absorbable sutures. All cases were performed without the use of an esophageal bougie.

After the surgery, a barium swallow was performed and when unsuspicious patients received an unrestricted diet to avoid the development of dysphagia due to scar tissue surrounding the device. After at least one overnight stay, patients were discharged from the hospital once they were eating solid foods.

### Statistical analysis

Statistical analysis was performed using SPSS® statistics 20.0 (IBM, Armonk, NY). Data were described using median (interquartile range) or mean (range). Statistical analysis appropriate for non-parametric data was used. Categorical variables were assessed using the Fisher exact test and continuous data using the Wilcoxon Rank test as appropriate. Comparison of multiple groups were done using ANOVA. Statistical significance was defined as a *p*-value < 0.05.

### Data availability

The datasets generated during and/or analyzed during the current study are available from the corresponding author on reasonable request

## Results

A total of 68 GERD patients (46 males, 22 females) underwent magnetic sphincter augmentation (MSA) at a median age of 45 years (IQR, 38–58). There were no significant differences in age, gender distribution and preoperative BMI between groups. Thirteen of the included patients (19%) were obese with a BMI ≥ 30 (range, 30–43.5). Three patients (4%) showed an ineffective esophageal motility (IEM) on high-resolution manometry. Demographics and results of preoperative diagnostics are shown in Table 1. A hiatal hernia was present in 52 patients and was repaired during the LINX implantation in 26 patients (50%). The median size of hiatal hernia was similar in patients that underwent hiatal repair [2 cm (IQR, 2–3)] and those who had exclusive MSA without crural repair [2 cm (IQR, 1–3)]. Accordingly, there were 26 patients in both, the NHR and the HR group (38% each) and 16 in the “no hiatal hernia” (NHH) group (24%). Severity of reflux disease based on preoperative pH-monitoring was comparable between groups.

**Table 1:** Demographic data and results of preoperative diagnostics.

Total n = 68 (100%)	NHH: n = 16 (24%)	NHR: n = 26 (38%)	HR: n = 26 (38%)	*p-*value
Sex (m vs. f)	11 (69) vs 5 (31)	16 (62) vs. 10 (38)	19 (73) vs. 7 (27)	0.670
Median age (IQR)	44 (38–54)	43 (32–49)	49 (40–59)	0.085
Presence of hiatal hernia	0 (0–0)	26 (100)	26 (100)	< .001
Median HH size in cm (IQR)	0 (0–0)	2 (1–3)	2 (2–3)	0.006
Median BMI*(IQR)	26 (23–29)	25 (23–29)	25 (22–29)	0.857
Median total # reflux episodes (normal < 73)	64 (52–75)	60 (43–84)	77 (56–94)	0.409
Median total percentage time pH < 4 (normal < 4.2%)	4.4 (3.6–9.2)	4.3 (1.5–11)	5.2 (2.7–9.8)	0.597
High-resolution manometry				0.491
Normal motility	16 (100)	25 (96)	24 (92)	
IEM^†^	0 (0)	1 (4)	2 (8)	
Median LES resting pressure (normal: 10–45 mmHg)	20 (15–26)	14 (8–24)	20 (14–27)	0.731
Median IRP^‡^ (normal < 20 mmHg)	9 (8–11)	7 (6–10)	8 (6–14)	0.063
Median GERD-HRQL total score^§^	26 (10–33)	28 (23–33)	17 (8–23)	0.005
**Symptoms:**				
Heartburn	15 (94)	25 (96)	25 (96)	0.491
Regurgitations	11 (69)	18 (69)	17 (65)	0.951
Difficulty swallowing	2 (13)	7 (27)	1 (4)	0.061

Error analysis: standard deviation.

Numbers provided in tables are medians (IQR). *BMI = Body Mass Index; ^†^IEM = ineffective esophageal motility; ^‡^IRP = integrated relaxation pressure of LES; ^§^GER-HRQL

total score ranges from 0–50.

Most common symptoms prior to surgery were heartburn (HR and NHR: 96%, NHH: 94; p = 0.491), regurgitations (HR: 65%, NHR and NHH: 69%, *p* = 0.951) and dysphagia (HR: 4%, NHR: 27%, NHH: 13%; *p* = 0.061). The latter was mostly due to esophagitis.

The median OR time was significant longer in HR patients [32 min (IQR, 26–39) vs. NHR: 25 min (IQR, 20–29) vs. NHH: 25 (IQR, 20–30); min; *p* = 0.008). No perioperative complications occurred. The device size most frequently used was 15 (12–16) in all groups. Patients after hiatal closure had a longer hospital stay (HR: 70% vs. NHR: 15% vs. NHH: 44% discharge after > 48 hours).

The median follow up time was 13 months (IQR, 4.2–45). Two patients (3%; 1 NHR and 1 NHH patient) with persistent dysphagia required endoscopic dilation. Procedures were performed 1 month and 7 months post-LINX implantation, respectively. Both patients underwent one-time balloon dilation (20 mm balloon) under x-ray control and were asymptomatic afterwards. The device was explanted in one HR and one NHR patient (3%) due to retrosternal pain and discomfort, respectively.

Postoperative esophageal functioning testing (EFT) were performed in 10 HR (38%), 15 NHR (58%) and 9 (56%) NHH patients (Table 2). Negative pH results were revealed in 80% of HR, 73% of NHR and 89% of NHH patients (*p* = 0.659). Surgery did not lead to relevant changes in BMI.

**Table 2:** Results of postoperative esophageal functioning testing (EFT).

Total n = 34 (100%)	NHH: n = 9 (27%)	NHR: n = 15 (44%)	HR: n = 10 (29%)	*p-*value
Time surgery to EFT (months)	12 (6–42)	43 (IQR, 33–50)	11 (IQR, 7–15)	0.003
Negative pH result	8 (89)	11 (73)	8 (80)	0.659
Median total # reflux episodes (normal < 73)	31 (20–39)	32 (IQR, 22–39)	37 (IQR, 32–70)	0.264
Median total percent time pH < 4 (normal < 4.2%)	0.9 (0.2–1.7)	1.2 (IQR, 0.5–4.9)	0.8 (IQR, 0.3–3.8)	0.749
High-resolution manometry				0.531
Normal esophageal motility	9 (100)	13 (87)	9 (90)	
IEM*	0 (0)	2 (13)	1 (10)	
Median LES resting pressure (normal: 10–45 mmHg)	23 (21–30)	23 (IQR,16–26)	22 (IQR, 14–25)	0.794
Median IRP^†^ (normal < 20 mmHg)	17 (12–24)	15 (IQR, 12–19)	13 (IQR, 12–14)	0.187

Error analysis: standard deviation.

Numbers provided in tables are medians (IQR). *IEM = ineffective esophageal motility;^**†**^IRP = integrated relaxation pressure of LES.

**Figure 1:**
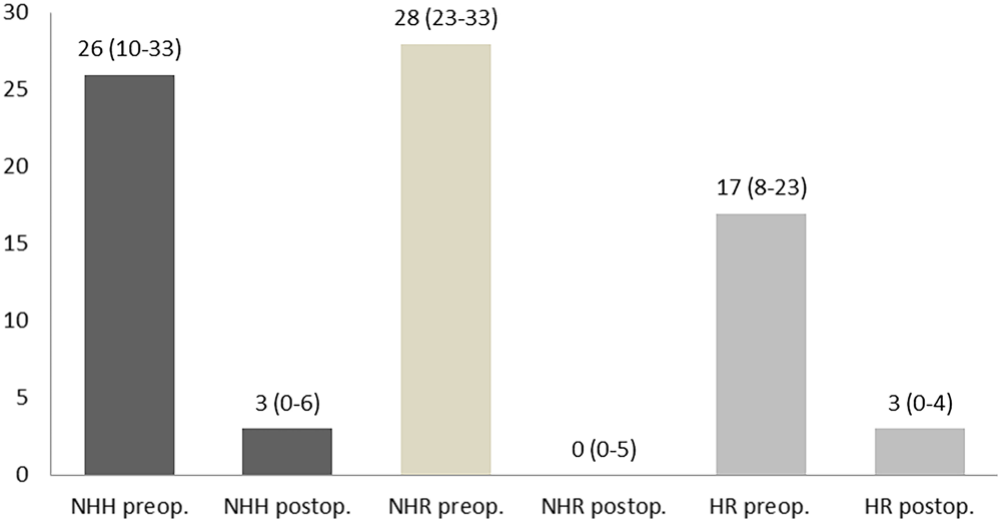
Comparison of pre- and postoperative GERD-HRQL total scores (median with IQR) between groups (NHH left, NHR group mid, HR group right); the maximum total score reachable is 50, with a lower score indicating a better QOL.

A standardized telephone interview was completed by 91% of patients (HR: n = 24 (92%); NHR: n = 22 (85%); NHH: n = 16 (100%). The postoperative GERD-HRQL total score was significantly reduced in all groups (HR: 17 vs. 3, NHR: 28 vs. 0, NHH: 26 vs. 3; *p* < 0.001) as shown in Fig. 1. Further, the median alimentary satisfaction (AS) was excellent with 8/10 in HR, 8/10 in NHH and 10/10 in NHR patients.

Satisfaction in terms of heartburn relief was achieved in 96%, 95% and 94% of HR, NHR and NHH patients (*p* = 0.953). Sphincter augmentation was rated more effective regarding heartburn relief than PPI use by 92%, 100% and 94% of the HR, NHR and NHH group (*p* = 0.402). Dependency on PPI intake was eliminated in approximately 87% of all groups. The overall postoperative outcome was rated excellent/good in 88% of HR/NHH patients and 91% of NHR patients (*p* = 0.921). Postoperative outcomes and Quality of Life results based on the surgical procedures are shown in Table 3.

**Table 3:** Postoperative outcomes and Quality of Life results based on surgical procedure.

Total n = 62 (100%)	NHH: n = 16 (26%)	NHR: n = 22 (35%)	HR: n = 24 (39%)	*p-*value
Median GERD-HRQL total score*	3 (IQR, 0–6)	0 (IQR, 0–5)	3 (IQR, 0–4)	0.798
Median Alimentary Satisfaction (AS)	8/10	10/10	8/10	0.276
Satisfaction with Heartburn relief	15 (94)	21 (95)	23 (96)	0.953
Heartburn relief by MSA better than with PPIs^†^	15 (94)	22 (100)	22 (92)	0.402
Postop. outcome rated excellent/good	14 (88)	20 (91)	21 (88)	0.921
Postoperative PPI use^†^	2 (13)	3 (14)	3 (13)	0.992
Postoperative BMI^‡^	26 (24–29)	26 (IQR, 23–28)	25 (IQR, 22–29)	0.926

Error analysis: standard deviation.

Numbers provided in tables are medians (IQR). *GERD-HRQL total score ranges from 0–50; ^†^PPI(s) = Proton pump inhibitors; ^‡^BMI: Body Mass Index.

**Figure 2:**
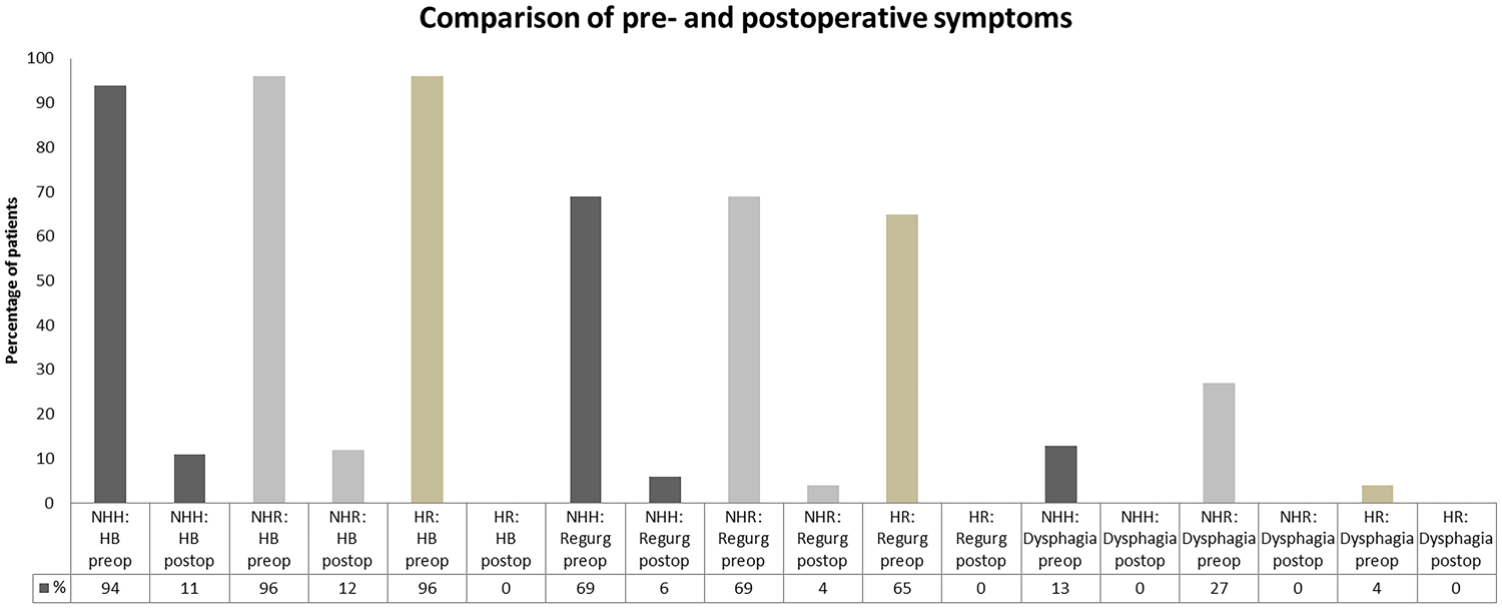
Comparison of pre- and postoperative symptoms (%) between groups (NHH, NHR and HR groups).

**Figure 3:**
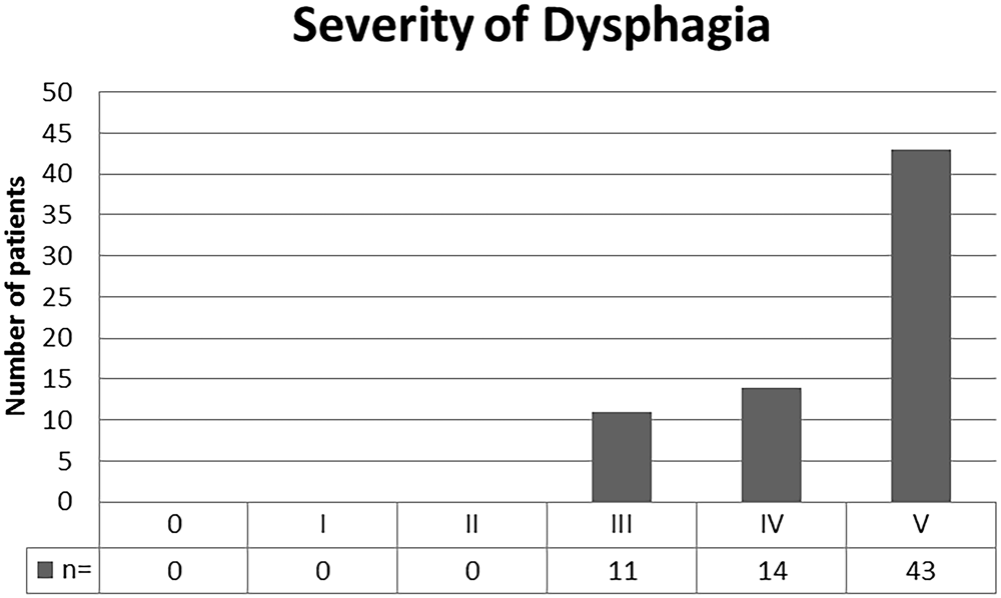
Frequency and degree of postoperative dysphagia based on the classification of Saeed *et al*.^14^. Columns from left to right: 0 = Unable to swallow (0%) I = Swallowing liquids with difficulty, solids impossible (0%) II = Swallowing liquids without difficulty, solids impossible (0%) III = Occasionally difficulty swallowing with solids (16%) IV = Rarely difficulty swallowing with solids (21%) V = Swallowing normally (63%).

Heartburn and regurgitations were eliminated in 100% of HR, 88% and 94% of NHR and 87% and 91% of NHH patients, respectively. There were no cases of persistent dysphagia (Table 4). A comparison of pre- and postoperative symptoms between groups is shown in Fig. 2. Rare difficulties swallowing solids was reported by 21% of patients (n = 14) while 16% (n = 11) of patients stated to have occasionally difficulties with swallowing solids. The frequency and severity of postoperative dysphagia based on the classification of Saeed *et al*. is shown in Fig. 3.

**Table 4:** Postoperative symptom relief based on surgical procedure.

Total n = 68 (100%)	NHH: n = 16 (24%)	NHR: n = 26 (38%)	HR: n = 26 (38%)	*p-*value
**Symptom relief**				
Heartburn (HB)	13/15 (87)	22/25 (88)	25/25 (100)	0.182
Regurgitations	10/11 (91)	17/18 (94)	17/17 (100)	0.489
Difficulty swallowing	2/2 (100)	7/7 (100)	1/1 (100)	1

Error analysis: standard deviation.

Numbers provided in tables are medians (IQR).

## Discussion

To date, no clear consensus exists on whether a full hiatal dissection and suture hiatal closure should be performed as a standardized step of magnetic sphincter augmentation. This decision is at the surgeon’s discretion and usually depends on the intraoperative presence and size of a hiatal hernia^2,4,18,19^. One advantage of focused over full hiatal dissection is that the hiatal/gastric anatomy remains intact and therefore makes a reoperation, if necessary, uncomplicated^20^. On the contrary, crural closure as a routine step of laparoscopic anti-reflux surgery is associated with lower postoperative rates of hiatal herniation and possibly better reflux control but may result in a higher incidence of dysphagia^9^.

Former studies presenting outcomes of magnetic sphincter augmentation mostly included both, patients after exclusive MSA and those with additional hiatal repair, thus, making it difficult to evaluate the impact of cruroplasty. In the current study, a consecutive series of 37 initial patients, treated with exclusive MSA, was followed by 31 patients that had undergone hiatal repair. Patients were classified into 3 groups according to the presence of a hiatal hernia and performed procedure (HR vs NHR).

The major finding of this study was a trend toward better reflux control and GERD symptom relief after crural closure compared to exclusive MSA in patients with a hiatal hernia. Furthermore, crural closure did not lead to increased dysphagia rates. Due to the relatively small sample sizes differences between groups did not reach statistical significance. While both surgical techniques led to satisfying outcomes, crural closure resulted in a higher rate of negative postoperative pH-results than exclusive MSA. This is comparable to the findings of Bonavina *et al*. who reported a negative pH rate of 75% five years after magnetic sphincter augmentation^6^.

Comparing MSA to laparoscopic Nissen fundoplication (LNF), previous studies found a more efficient reduction of the baseline percentage time pH < 4 following LNF than after MSA^21^. Further, LNF was reportedly leading to significant lower acid exposure of the distal esophagus and PPI intake than MSA^19,21^. This might be due to the impact of hiatal closure in LNF patients that was not performed in the majority of MSA patients.

Louie *et al*. described in 2013 that the hiatal repair may not only be as important as the LES reinforcement in reflux control, as was shown in 1971 by Woodward *et al*., but also contributes more to the LES pressure, than a Nissen fundoplication does^8,22,23^.

The 100% rate of heartburn elimination after crural closure in this series exceeds both, the one following exclusive MSA and the previously reported 94% of heartburn relief after LINX implantation^21^. Indeed, this high percentage of heartburn relief matches the rate of 96.5% found after LNF^21^. Satisfaction rates regarding overall outcome and heartburn relief were comparable between groups in the recent study. This is consistent with the fact that other studies did not find significant differences in overall satisfaction rates comparing MSA with LNF^19^.

The rate of elimination of regurgitations varied between groups and ranged from 91%-100%. These numbers are acceptable considering that LNF usually leads to elimination in 94%^21^. However, other studies described elimination of regurgitations in 100% (n = 28) after MSA^21^.

The percentage of patients that could eliminate their PPI use was comparable between procedures. Intake of acid suppression medication ranges between 8%-20% after MSA and 8–14% after LNF^1,2,4,6,8,18,19^. With 13% of HR patients using PPIs in this series, crural closure in MSA patients appears to be leading to results as those after laparoscopic fundoplication.

The significantly decreased GERD-HRQL total score after surgery in all 3 groups indicates a significant increase of GERD-specific quality of life after MSA. This matches the observations of Bonavina *et al*. who reported a significant reduction of the mean GERD-HRQL total score from 25.7 to 3.3 four years after MSA.

The current study showed excellent alimentary satisfaction ranging from 8–10 out of possible 10, indicating that patients are comfortable after LINX implantation.

No perioperative complications occurred in this series while rates of postoperative interventions and device removal were low and comparable with those published previously. In the evaluation phase of LINX, reflected by the pivotal trial published in 2013, 19 out of 100 patients underwent dilatation due to dysphagia after MSA^6,24^. Bonavina *et al*. described a dilation rate of only 2%^25^. Device explantation reportedly ranges from 2% to 7%^18,25^.

There were no cases of persistent dysphagia in this study which suggests that crural closure does not result in higher postoperative dysphagia rates than selective sphincter augmentation. Mild dysphagia rates after MSA have been described to vary between 5%-45% while persistent dysphagia usually does not occur at the last follow up^16,18,25,26^.

There were some limitations to this study including its retrospective nature. The concept of comparing 2 consecutive series of different surgical techniques poses the problem of varying follow-up times. Further, postoperative objective testing was not performed uniformly at a specific follow-up time resulting in different long “surgery to EFT”- times in NHR and HR groups. Hence, the comparison of outcomes and postoperative EFT results between groups might be biased. Acknowledging these concerns, we have initiated to schedule all LINX patients for objective testing at the 1-year follow-up visit. We intend to continue reporting outcomes and to evaluate if results change with longer follow-up. The relatively small sample sizes of groups prevented differences from reaching statistical significance. However, our findings add important information on the impact of crural closure during magnetic sphincter augmentation on reflux control. Comparing the two surgical procedures (MSA vs. MSA with crural closure), there was a clear trend of crural closure resulting in superior reflux control and gastrointestinal symptom relief without increasing dysphagia rates. Further prospective trials with larger sample sizes are necessary to confirm the advantages of hiatal repair over exclusive magnetic sphincter augmentation in GERD patients with hiatal hernia.

## Conclusion

Magnetic LES augmentation leads to significant gastrointestinal symptom relief, increased GERD-specific quality of life and good alimentary satisfaction with low perioperative morbidity rates. In patients with hiatal hernia, crural closure tends to result in better reflux control and GERD symptom relief compared to exclusive MSA without increasing postoperative dysphagia rates. Further studies with larger sample sizes are necessary to confirm these findings. Hiatal repair should be considered as routine step of magnetic sphincter augmentation in GERD patients with hiatal hernia.

## Electronic supplementary material

Supplementary Information
