# Supplementary material for: Crural Closure improves Outcomes of Magnetic Sphincter Augmentation in GERD patients with Hiatal Hernia
Source: Sci Rep. 2018 May 9;8:7319. doi: 10.1038/s41598-018-24322-1 (PMC5943411; doi:10.1038/s41598-018-24322-1)
Supplement: Supplementary file 1 — Supplementary Information [file 41598_2018_24322_MOESM1_ESM.pdf]

Crural Closure improves Outcomes of Magnetic Sphincter Augmentation in GERD patients with Hiatal Hernia

Dr. Katrin Schwameis
Dr. Milena Nikolic
Dr. Deivis G. Morales Castellano
Ariane Steindl
Sarah Macheck
Univ. Prof.Dr. Riegler M.
Dr. Ivan Kristo
Dr. Barbara Zörner
Univ.Prof.Dr. Sebastian F. Schoppmann

Department of Surgery, Division of General Surgery, Medical University of Vienna
Waehringer Guertel 18-20, 1090 Vienna, Austria

Table of contents

1. Background 2
2. Aim 3
3. Patients 3
4. Outcomes 3
   4.1 Main outcomes 3
   4.2 Secondary outcomes 3/4
5. Methodology 4
6. Statistics 4
7. Data security 4
8. Benefit risk evaluation 4
9. References 4/5

1. Background

Gastroesophageal Reflux Disease (GERD) is one of the most common gastrointestinal disorders affecting up to 25% of the population in the Western World.[1] The surgical gold standard in the treatment of GERD is the laparoscopic fundoplication. [1-3] Typical side effects include gas bloat syndrome, a reduced ability to vomit and belch and dysphagia. A further deterrent of this procedure is the extensive alteration of the anatomy making it difficult to re-operate if needed. [4] A recently introduced alternative with possibly less side effects is the magnetic sphincter augmentation (MSA) with a small device consistent of magnetic beats (LINX® Reflux Management System; Torax Medical, Maple Grove, MN). The device is placed laparoscopically around the gastroesophageal junction to augment the barrier function of the lower esophageal sphincter (LES), suppressing reflux episodes while enabling the physiological functions of the LES uninhibited. This can be accomplished with either focused or full dissection.[5] The former minimally invasive procedure keeps the possibility of further anti-reflux surgery open, if needed, by maintaining the esophageal and hiatal anatomy.[4,6]

Former studies showed that the implantation of LINX was safe and efficient in reducing the dependence on proton pump inhibitors (PPIs), improving GERD-specific quality of life while leading to a low rate of side effects. [3,6,7] While MSA was initially limited to patients with small or no hiatal hernias Rona K. et al recently reported their encouraging outcomes of MSA in patients with hiatal hernias up to 7cm. They showed that these patients had similar postoperative symptom relief, decreased PPI requirement, GERD-HRQL scores and dysphagia rates as patients with smaller hernias. [8]

As described previously hiatal dissection followed by suture crural repair is a crucial step in the process of laparoscopic anti-reflux surgeries to prevent hiatal herniation.[9]

However, no consensus exists on whether additional hiatal repair should be routinely performed in patients undergoing sphincter augmentation. Currently, to our knowledge, no study has been published comparing outcomes of patients after exclusive MSA with those who have had MSA and additional crural closure.

2. Aim

The aim of this study is to assess if hiatal repair in MSA patients significantly influences postoperative outcomes including reflux control and dysphagia rate.

3. Patients

Retrospective review of all GERD patients that have undergone magnetic sphincter augmentation (MSA) between 03/2012 and 03/2017 at our institution. Demographic and clinical data will be reviewed and analyzed.

4. Outcomes

4.1. Main outcome

Level of reflux control provided by magnetic sphincter augmentation (MSA)

Comparison of postoperative outcomes in patients with MSA vs. those with MSA and crural closure

4.2. Secondary outcomes

Quality of Life after sphincter augmentation in GERD patients

5. Methodology

Analysis taking place at the Department of Surgery, Medical University of Vienna, AKH Vienna

6. Statistics

Statistical analyses are performed using SPSS® statistics 20.0 (IBM, Armonk, NY). Data are described using median (interquartile range) or mean (range). Statistical analysis appropriate for non-parametric data will be used. Categorical variables will be assessed using the Fisher exact test and continuous data using the Wilcoxon Rank test as appropriate. Comparison of multiple groups will be done using ANOVA. Statistical significance is defined as a *p*-value < 0.05.

7. Data security

All Patients are consecutively numbered and pseudonymizated prior to analyses. Only authorized persons can access data. Data are collected at a Laptop with security access at the Division of General Surgery

8. Benefit risk evaluation

Patients have no direct benefit from that study. The only risk, revealing of sensible data, is eliminated thorugh pseudonymization and limited access. The results of this study can be used for hypothesis generation of further studies.

9. References

1 Reynolds, J. L. *et al.* Charges, outcomes, and complications: a comparison of magnetic sphincter augmentation versus laparoscopic Nissen fundoplication for

the treatment of GERD. *Surgical endoscopy* **30**, 3225-3230, doi:10.1007/s00464-015-4635-6 (2016).
2 Reynolds, J. L. *et al.* Laparoscopic Magnetic Sphincter Augmentation vs Laparoscopic Nissen Fundoplication: A Matched-Pair Analysis of 100 Patients. *Journal of the American College of Surgeons* **221**, 123-128, doi:10.1016/j.jamcollsurg.2015.02.025 (2015).
3 Rona, K. A. *et al.* Efficacy of magnetic sphincter augmentation in patients with large hiatal hernias. *Surg Endosc*, doi:10.1007/s00464-016-5204-3 (2016).
4 Czosnyka, N. M. *et al.* Outcomes of magnetic sphincter augmentation - A community hospital perspective. *Am J Surg*, doi:10.1016/j.amjsurg.2016.09.044 (2016).
5 DeMeester, T. R. Surgical Options for the Treatment of Gastroesophageal Reflux Disease. *Gastroenterology & hepatology* **13**, 128-129 (2017).
6 Bonavina, L., Saino, G., Lipham, J. C. & Demeester, T. R. LINX((R)) Reflux Management System in chronic gastroesophageal reflux: a novel effective technology for restoring the natural barrier to reflux. *Therapeutic advances in gastroenterology* **6**, 261-268, doi:10.1177/1756283X13486311 (2013).
7 Skubleny, D. *et al.* LINX(R) magnetic esophageal sphincter augmentation versus Nissen fundoplication for gastroesophageal reflux disease: a systematic review and meta-analysis. *Surg Endosc*, doi:10.1007/s00464-016-5370-3 (2016).
8 Rona, K. A. *et al.* Efficacy of magnetic sphincter augmentation in patients with large hiatal hernias. *Surgical endoscopy* **31**, 2096-2102, doi:10.1007/s00464-016-5204-3 (2017).
9 Watson, D. I., Jamieson, G. G., Devitt, P. G., Mitchell, P. C. & Game, P. A. Paraoesophageal hiatus hernia: an important complication of laparoscopic Nissen fundoplication. *The British journal of surgery* **82**, 521-523 (1995).
